# The Energy Landscapes of Repeat-Containing Proteins: Topology, Cooperativity, and the Folding Funnels of One-Dimensional Architectures

**DOI:** 10.1371/journal.pcbi.1000070

**Published:** 2008-05-16

**Authors:** Diego U. Ferreiro, Aleksandra M. Walczak, Elizabeth A. Komives, Peter G. Wolynes

**Affiliations:** 1 Department of Chemistry and Biochemistry, University of California San Diego, La Jolla, California, United States of America; 2 Center for Theoretical Biological Physics, University of California San Diego, La Jolla, California, United States of America; 3 Department of Physics, University of California San Diego, La Jolla, California, United States of America; UCSF, United States of America

## Abstract

Repeat-proteins are made up of near repetitions of 20– to 40–amino acid stretches. These polypeptides usually fold up into non-globular, elongated architectures that are stabilized by the interactions within each repeat and those between adjacent repeats, but that lack contacts between residues distant in sequence. The inherent symmetries both in primary sequence and three-dimensional structure are reflected in a folding landscape that may be analyzed as a quasi–one-dimensional problem. We present a general description of repeat-protein energy landscapes based on a formal Ising-like treatment of the elementary interaction energetics in and between foldons, whose collective ensemble are treated as spin variables. The overall folding properties of a complete “domain” (the stability and cooperativity of the repeating array) can be derived from this microscopic description. The one-dimensional nature of the model implies there are simple relations for the experimental observables: folding free-energy (ΔG_water_) and the cooperativity of denaturation (*m*-value), which do not ordinarily apply for globular proteins. We show how the parameters for the “coarse-grained” description in terms of foldon spin variables can be extracted from more detailed folding simulations on perfectly funneled landscapes. To illustrate the ideas, we present a case-study of a family of tetratricopeptide (TPR) repeat proteins and quantitatively relate the results to the experimentally observed folding transitions. Based on the dramatic effect that single point mutations exert on the experimentally observed folding behavior, we speculate that natural repeat proteins are “poised” at particular ratios of inter- and intra-element interaction energetics that allow them to readily undergo structural transitions in physiologically relevant conditions, which may be intrinsically related to their biological functions.

## Author Summary

Repeat-proteins are coded in repetitions of similar amino acid stretches. Unlike typical globular domains, repeat-protein domains fold into elongated superhelical shapes of stacked elements, stabilized only by interactions within each repeat or between adjacent repeats. This architecture allows folding to be treated as a quasi–one-dimensional problem. We introduce an analytical model that describes the folding energy landscape of repeat-proteins, based on a representation in terms of spin variables. This representation groups together conformations on the basis of the degree of order in local quasi-independent folding units, often called foldons. We derive simple relations between the experimentally observed stability and cooperativity of denaturation of the whole repeat-domain, which differ from those found in three-dimensionally connected globular proteins. Folding simulations on perfectly funneled landscapes reproduce these relations. We document that these relations are experimentally observed in a variety of repeat-protein systems. We show the parameters in the foldon spin description can be predicted on the basis, largely, of protein topology, reflecting the funneled energy landscape.

## Introduction

Many proteins are nearly periodic. Instead of being formed by ‘apparently random’ amino acid sequences [1], repeat-proteins are made up of tandem arrays of similar stretches, usually between 20 and 40 amino acids in length [2]. In ‘physiological’ conditions, these polypeptides fold up into elongated architectures of repeating structural motifs that stack one upon the next producing extended superhelical structures ([2] and references therein). Quasi-one dimensional, these non-globular folds are stabilized only by interactions within each repeat or between adjacent repeats. In general there are no obvious contacts between residues distant in sequence. This seemingly simple architecture contrasts with typical globular domains in which the polypeptide chain ‘wraps around’ to form multiple sequence-distant interactions. For globular proteins these sequence-distant interactions often play critical roles in folding kinetics, and dictate the overall topology of the protein [3],[4]. Repeat proteins, by virtue of their inherent symmetries both in primary sequence and three dimensional structure, should have an underlying folding landscape reflecting these symmetries. The near periodicity of repeat proteins allows a general description of their energy landscape which can help us appreciate their biological function.

One natural way to model repeat-protein folding is to coarse-grain the description of the protein architecture to be that of a linear array of elementary ‘units’ that interact locally with each other. Though each unit in such an array is complex, their simplification yields a description of the low free energy ensembles as corresponding to those of a classical one-dimensional Ising magnet [5]. In Ising models, each site is taken to be in one of two states (i.e.: spin up/spin down; helix/coil, folded/unfolded). Interaction energies are defined only locally between nearest neighbors. Owing to its one dimensional connectivity, the thermodynamic properties of the system can be computed exactly. More than half a century ago, Zimm and Bragg used this Ising description to describe the formation of α-helices [6]. In their model each residue was considered to be able to be ordered in a helical state of low energy, or be in a coil state of high energy, reflecting an ensemble of possible chain orientations. Recently similar models have been used to describe repeat-protein folding [7]–[9]. In this case each repeating segment is taken to be ordered in a low energy state or to populate a disordered many dimensional ensemble of high entropy. Clearly this description lumps together many different substates in which a repeating unit is only partially ordered. In simpler but somewhat less precise terms, one might say that we approximate each element as folding as a highly cooperative unit. The hope is that in some sense other high free energy states are sufficiently rare as to be unnoticed in the thermal ensemble. In what sense is this description adequate for the low free energy ensemble? Also, can we discover the appropriate units to think of as quasi-independent? Finding such units clearly is related to the question of the existence of foldons [10], autonomously folding units which can serve as ‘building blocks’ of protein structures [11].

The most extensive investigations on the folding of repeat-proteins have focused on the ankyrin (ANK) and the tetratricopeptide (TPR) repeat protein families. Most of the natural members of these families are composed of 4 to 10 repeats, but sequences coding up to ∼50 repeats have been found [12]. The shorter members with 2 to 7 repeats have been very carefully characterized in terms of their experimental folding behavior. Folding repeat-proteins of these lengths appears to follow a two-state transition, in which only the fully denatured ensemble and a nearly unique folded state are significantly populated at equilibrium ([13],[14] and references therein). The experiments along with simulations based on perfectly funneled landscapes suggest that this cooperative behavior can be understood if the domains fold up by a mechanism that is reminiscent of nucleation-propagation growth [8],[15]. Once an initial nucleation takes place, the individual structural modules of the repeat-protein serially fold up in a highly cooperative fashion that results in the folding of the complete ‘domain’. Apparently, as in the helix-coil transition, the intrinsic stability of the folding elements is low compared to the free energy of stabilization from forming an ‘interface’ between neighbors [8],[15]. In the helix-coil transition, the Pauling scheme requires four hydrogen bonds to form an α-helix turn, resulting in a large nucleation free energy that comes from the necessity to properly configuring several successive dihedral angles in a subunit, losing their entropy before hydrogen bond stabilization is gained. In the case of repeat-proteins, because of the delicate balance in each subunit, subtle variations in the interactions in and between the repeats give the impression of major changes in the folding landscape [16],[17]. Such variations may then ‘decouple’ the folding of the elements. This balance implies that for sufficiently long repeating arrays partially folded species become populated. These species may be characterized at equilibrium [18]–[20]. In a sense they provide snapshots of ensembles closely resembling the fleetingly formed transition state ensemble for globular proteins, providing information about the fine structure of the energy landscape, about its symmetry and deviations from it [16],[17].

The energy landscape theory of protein folding is based on the ‘principle of minimal frustration’ [21]. This principle states that the energy of the protein decreases more than what may be expected by chance as the protein assumes conformations progressively more like the ground (native) state. In other words, there is a strong energetic bias towards the native basin that overcomes both the asperities of the landscape and ultimately the entropy of the chain. The resulting overall landscape picture is that of a rough funnel [22]. When energetic frustration is low enough, the native energy and folding entropy primarily compete. Since these mainly depend on protein topology, topology becomes a key factor governing folding reactions. It has been shown that the structures of transition state ensembles [23],[24], the existence of folding intermediates [25], dimerization mechanisms [26], and domain swapping events [27] are often well predicted in models where energetic frustration has been removed and topological information of the native state is the sole input. Still, inhomogeneity in the native contacts energetics, non-native interactions and the residual local frustration present in the native ensemble do contribute to the functional characteristics of proteins, ‘molding’ the roughness that underlies the detailed protein dynamics [28],[29].

The internal symmetries of repeat-proteins suggest that the overall folding properties of a complete ‘domain’ (the stability and cooperativity of the array) may be derived from a microscopic description of the energy balance within each folding element and its interactions with its neighbors. Furthermore, the minimal frustration principle suggests that “on average” these interactions can be inferred from knowledge of the protein topology. However, the paucity of contacts in interfaces can give significant fluctuations.

Here, we study how an ‘Ising-like’ description in terms of foldon spin variables for the repeat-containing proteins can be obtained from a more fundamental model based on perfectly funneled energy landscapes. The parameters of the description follow to a first approximation from the protein topology. By explicitly transforming from this more detailed model, we show how an Ising foldon-spin description for the low free energy states emerges and ultimately leads to the ability to predict the global folding properties of repeat-proteins. For illustration we show how the parameters in the Ising description of the energy landscape of a family of TPR proteins can be extracted from the elementary interactions between residues. We find that the parameters in the Ising model, like kinetic barriers for folding globular proteins, are most strongly a property predictable from native state topology, although again subtle but important changes can be made by tuning the sequence. We must emphasize that the general structure of the energy landscapes of proteins are robust, but the details of the kinetic routes taken through it depend on smaller energy scales, associated with inhomogeneities. Small perturbations may therefore re-route the transitions [30]. The one-dimensional physics where a single defect can interrupt the development of other stabilizing interactions, makes the macroscopic ensembles more sensitive to local details than one usually finds for the folding of the three dimensionally connected globular proteins, where sequence tuning may be less important for geometrically correct overall folding, but may be critical for function [28],[29].

## Results/Discussion

### Analytical Repeat-Protein Folding Model

#### Definitions

Repeat proteins may be thought of as a linear array of structural elements. The low free energy ensembles of these arrays may be represented as the states of a collection of elementary folding units. Each unit can assume two macro-states: folded (F) or unfolded (U). Both macrostates represent ensembles of atomic level structures. Interaction between nearest neighbors are most important and depend on whether each unit can be considered to be folded or not. The two states can be thought of as foldon spin variables. Hence the free energy of the bulk of the thermally occupied states can be described with a coarse-grained one-dimensional Ising model having a Hamiltonian:
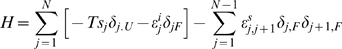
(1)


An unfolded subunit contributes a low free energy because of the entropy of its available configurations, *s_j_*. If the unit is not too compact we can take its internal energy to be zero. On the other hand, folded elements have little entropy but have an internal folding free energy (averaged over the solvent) of 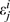. In the coarse-grained description, the interaction energies between neighboring elements are zero when either of the elements is unfolded, but is equal to a surface energy 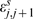 if both elements are folded. Since the systems we want to treat are linear and finite, the end elements (or ‘caps’ [31]) must be explicitly treated as having only one neighbor. The influence of a chemical denaturant can be modeled as binding with the unfolded elements. This influence can be described as an increase in the entropy of the system, by allowing the protein to access a greater configurational space, *s_j_* = *s*_0,*j*_+α*_j_x_j_*, where *x_j_* is the denaturant concentration [i.e. urea, guanidine], *s*_0,*j*_ the accessible entropy without denaturant. α*_j_* is a denaturation parameter that describes the susceptibility of a given element to interact with the denaturant [7]. Within this model a protein can unfold both as a result of an increase in temperature (*T* = 1/β), or by an increase in the concentration of denaturant *x_j_*.

#### Derivations

At the level of most experiments, the thermodynamics for folding of a repeat protein is usually quantified by following a spectroscopic signal averaged over the sample. Often one quotes the overall change in free energy between the macroscopic thermodynamic states in the absence of chemical denaturant (ΔG_water_) or the folding temperature (T_f_), along with a cooperativity parameter that describes how rapidly the free energy changes with denaturant (*m*). In case of a chemical denaturation, commonly one speaks of the cooperativity ‘*m*-value’ usually obtained assuming a linear extrapolation of the form 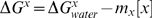 [32]. The *m*-values are what are called in thermal physics ‘susceptibilities’. These susceptibilities can be written in terms of correlation functions. For the present model, it is easy to show that the cooperativity parameter ‘*m*’ is directly related to the equilibrium correlation functions 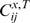 for the degree of order of the protein. The appropriate correlation functions are defined by:
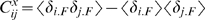
(2)if the protein is unfolded by chemical means. If the protein is unfolded by temperature, the relevant correlation function is:
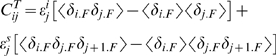
(3)The corresponding cooperativity parameters are:
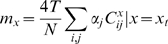
(4)

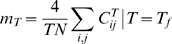
(5)where *x_t_* and *T_f_* are the midpoints of the folding transitions, concentration of denaturant and the folding temperature, respectively. Intuitively it is easy to see that when units further apart are more correlated in their macrostate occupation, the protein thermodynamically responds more strongly to changes in concentration of denaturant or temperature, i.e.: folding is more cooperative. The precise correlation function for temperature or chemical denaturation are different. A three-point correlation function enters for the susceptibility in temperature denaturation. Thus, the cooperativity of the transition will appear to be a more global phenomenon for temperature denaturations (see below).

When end-effects are neglected, i.e. for a translational invariant system (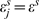, 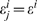, *s_j_* = *s*) having therefore many repeats, these expressions become:
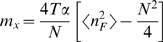
(6)

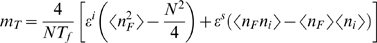
(7)where *n_F_* is the number of folded elements and *n_i_* is the number of folded elements that have folded elements as neighbors.

#### Folding mechanism

We first recall some analytical results for the case in which all repeating elements are identical and the protein consists of a large number of repeats. This is what physicists call a translationally invariant model with periodic boundary conditions in the large N limit. These yield rather simple expressions for the global folding susceptibility parameters. However, natural proteins usually have a relatively small number of repeats, so the large N analysis is complemented by numerical evaluations of proteins of finite length in the appropriate quantities for various regions of the parameter space.

#### Chemical denaturation

**Figure 1:**
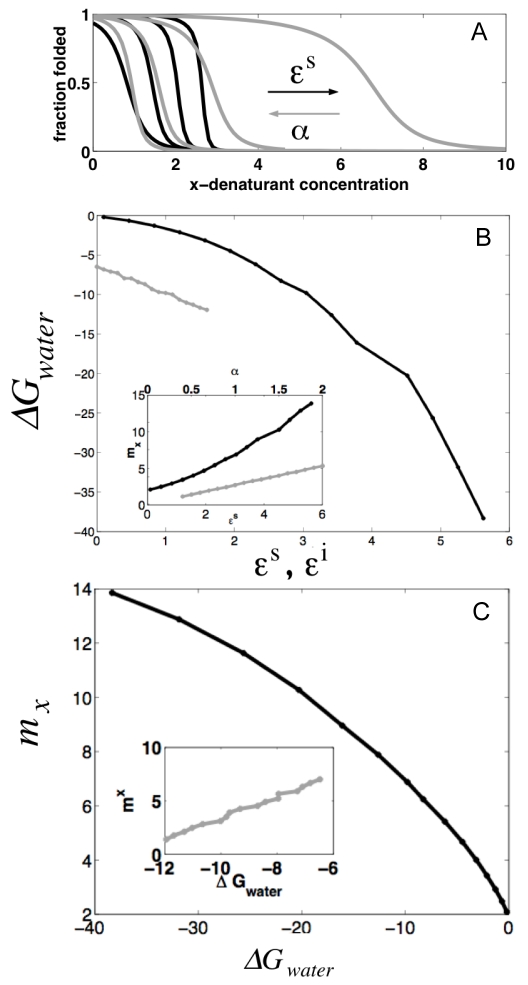
Numerical calculations of the analytical model for a finite protein of N = 14 undergoing chemical denaturation. (A) Fraction folded as a function of denaturant (*x*) at increasing ε*^S^* (ε*^i^* = 1.1, α = 2, *s_0_* = 1 fixed)(black lines) and simultaneously decreasing α and increasing ε*^i^* (ε*^S^* = 3, *s_0_* = 1 fixed) (gray lines). (B) Dependence of the free energy between the fully folded and the fully unfolded states (ΔG_water_) for the changes in parameters described in (A). Insert: cooperativity of the folding transition as a function of the varied parameters. (C) Relationship between ΔG_water_ and *m*-values for changes in parameters described in (A).

The analytical expression for the *m*-value in the large N limit is:
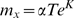
(8)where α is the susceptibility of a single element (*vide supra*) and 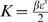. We see the protein susceptibility responds exponentially to changes in inter-repeat interaction energy. For finite systems with end-effects, the numerical evaluations also show that the global measurements on finite proteins should depend only slightly on the internal energetic parameters of the elements, *s*_0_ or ε*^i^*, but that they are sensitive to the energy of interaction between elements, ε*^S^*, which strongly affects them (Figure 1). The exponential dependence on the interaction energy is evident for small values of the interaction energy ε*^S^*, but for finite size proteins the *m*-value change saturates (Equation 9), once the correlation spans the whole protein. The protein cannot become any more cooperative. In the large N limit, the transition midpoint and the free energy in water are:
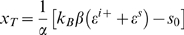
(9)

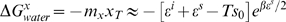
(10)


Figure 1 shows the dependence of the free energy and the apparent cooperativity of a protein of N = 14 units for various regions of the parameter space. Again, the protein becomes more stable and more cooperative as ε*^s^* increases. This result can be physically interpreted as arising from a bigger penalty for having an unsatisfied interface between elements, so that the protein behaves more as a single folding domain. As expected, the extrapolated stability of the protein (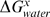) does not change with the capability of the particular residues to binding of the denaturant molecules (α). Only changing the interaction energy between elements results in a simultaneous change in the cooperativity and stability, revealing an intimate coupling between the two (Figure 1C). On the other hand, changing the properties internal to individual elements by perturbing α and ε*^i^* at the same time, also results in a roughly linear relationship between *m* and ΔG*_water_* (Figure 1C inset). However, in the latter case, the cooperativity decreases with increasing stability. The energy gain on folding the individual elements is enough to compensate the entropy cost of having an unsatisfied interface, the elements uncorrelate, and the cooperativity breaks down.

#### Thermal denaturation

The analytical expression for the appropriate susceptibility in the large N limit is:
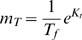
(11)where *K_t_* = *K*(*T_f_*), 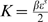 and the transition temperature (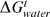) is:
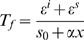
(12)


**Figure 2 pcbi-1000070-g002:**
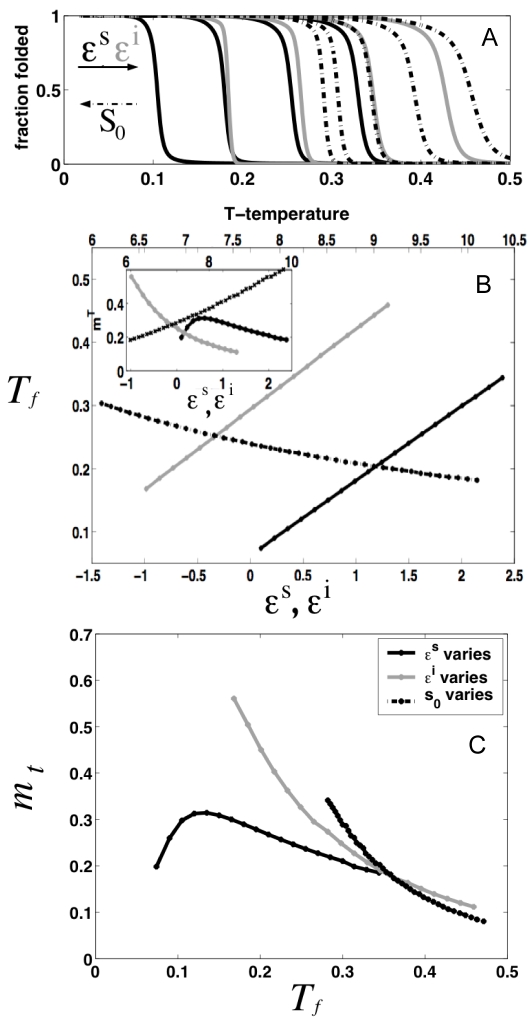
Numerical calculations of the analytical model for a finite protein of N = 14 undergoing thermal denaturation. (A) Fraction folded as a function of temperature (*T*) at increasing ε*^S^* (ε*^i^* = 0.5, α = 2, *s_0_* = 8 fixed)(black solid lines), increasing ε*^i^* (ε*^S^* = 2.5, *s_0_* = 8 fixed) (gray lines) and decreasing *s_0_* (ε*^S^* = 2.5, ε*^i^* = 0.5 fixed) (black dashed lines). (B) Dependence of the transition temperature between the fully folded and the fully unfolded states (*T_f_*) for the changes in parameters described in (A). Insert: cooperativity of the folding transition as a function of the varied parameters. (B) Relationship between *T_f_* and *m*-values for changes in parameters described in (A).

The stability of the protein increases linearly as the effective field (ε*^i^*+ε*^S^*) increases, and shows an inverse relationship with the entropy of the individual elements and chemical denaturant activity. This dependence is also seen in the numerical study for a protein having a small finite number of repeats (Figure 2). The cooperativity depends on a combination of three competing energy scales, the temperature and both the energy of formation of a single element unit ε*^i^* and of the interfaces ε*^S^*. If the inter-element interaction is lower than the internal energies, the cooperativity and the stability increase with ε*^S^* (Figure 2). Interestingly, when the interaction energy is higher than the internal energy balance within each element, strengthening the interaction between neighboring elements actually decreases the apparent cooperativity of the protein folding (Figure 2C). This counter-intuitive result is not found when monitoring chemical denaturations. The difference arises from the different forms of the correlation function 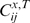 (Equation 3), entering the thermal denaturation susceptibilities. As the temperature is increased, the unfolding of certain elements results in the preferential unfolding of neighboring domains, since when an element is unfolded it has no means of interacting with its neighbor, and no possible surface energy gain. As ε*^S^* increases, the three body correlations are rare resulting in a decrease of the overall cooperativity of the system.

### The Effect of Mutations

**Figure 3:**
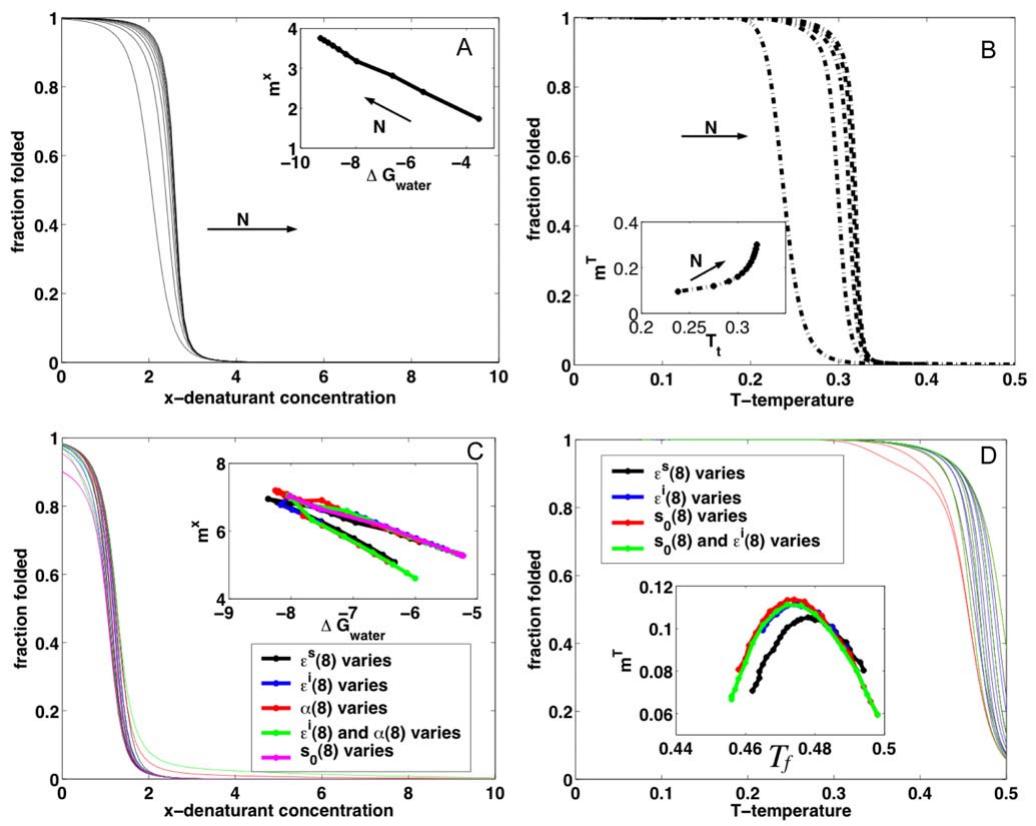
Effect of mutations on the coarse-grained model. (A) Fraction folded for chemical denaturation as a function of denaturant (*x*) for proteins of different length. Insert: Relationship between ΔG_water_ and *m*-values. (ε*^S^* = 1.4, ε*^i^* = 1.5, α = 2, *s_0_* = 2 fixed). (B) Fraction folded for temperature denaturation as a function of *T* for proteins of different length. Insert: Relationship between *T_f_* and *m*-values. (ε*^S^* = 2.1, ε*^i^* = 0.5, *x* = 0, *s_0_* = 8). (C) Fraction folded for chemical denaturation as a function of *x* for proteins with local perturbations in the eighth repeat as specified in the legend. Insert: Relationship between ΔG_water_ and *m*-values. (ε*^S^* = 3.0, ε*^i^* = 0.5, α = 2, *s_0_* = 1). (D) Fraction folded for temperature denaturation as a function of *T* for proteins with local perturbations in the eighth repeat specified in the legend. Insert: Relationship between *T_f_* and *m*-values (ε*^S^* = 2.5, ε*^i^* = 1.5, *x* = 0, *s_0_* = 8).

In contrast to the situation for globular proteins, repeat-based architecture allows large sequence deletions to be made without severely disrupting the overall fold [7],[8]. Such deletions correspond in the present model to a change in N, the number of folding elements. For a translationally invariant model with no end-effects, both the analytical results and the numerical calculations show that larger proteins exhibit both increased stability and cooperativity (Equations 6–7) (Figure 3A and 3B). This pattern directly results from the fact that more elements are present and that they interact with each other in the same way, thus more terms contribute to the correlation function. In the case of chemical denaturations, this results in a constant that leads to a linear *m*(ΔG*_water_*) function, as is experimentally observed (see below). For thermal denaturations the change in the susceptibility is low for shorter proteins and becomes exponential as N grows.

Depending on their location in the structure, point mutations can be modeled as local changes in the internal energy of a particular element, or the interaction with its neighbors, or both. We analyze these effects by numerical calculations of a N = 14 protein for which only the parameters for one element are perturbed, mimicking a site-directed mutation. If the energetic stabilization of the mutant element is smaller than that of the others, then both the cooperativity and the stability increase when the parameter of a given element increases (insets in Figure 3C and 3D). Small changes in either intra-element or inter-element energetics result in big overall effects. However, these changes reach a threshold when the local parameter becomes larger than those that describe the typical energy scales. In this case the protein does not behave as a single folding unit and ‘splits’ into folding domains, thus the overall cooperativity decreases (insets to Figure 3C and 3D). This can be interpreted as a global effect ‘felt’ by the whole protein, a situation that is more drastic when the number of repeating units is of the order of the correlation length of the system. In order to pin down the parameter regime for natural repeat-proteins, we now compare these general results for the one-dimensional Ising behavior to results obtained from molecular dynamics folding studies on perfectly funneled landscapes and to the experimentally determined folding mechanisms.

### Analyzing the Folding by Discrete Molecular Dynamics Simulations

We study a perfectly funneled folding model. In such a homogeneous model only a single energy scale enters so the behavior only depends on the topology and the ratio of *T* to *T_f_*. In the residue level representation a protein is treated as a series of connected beads located at the Cα position of each amino acid. Only residues which are in contact with each other in the native state are given a favorable energy, leading to a smooth and homogeneous funnel-like energy landscape [22].

**Figure 4:**
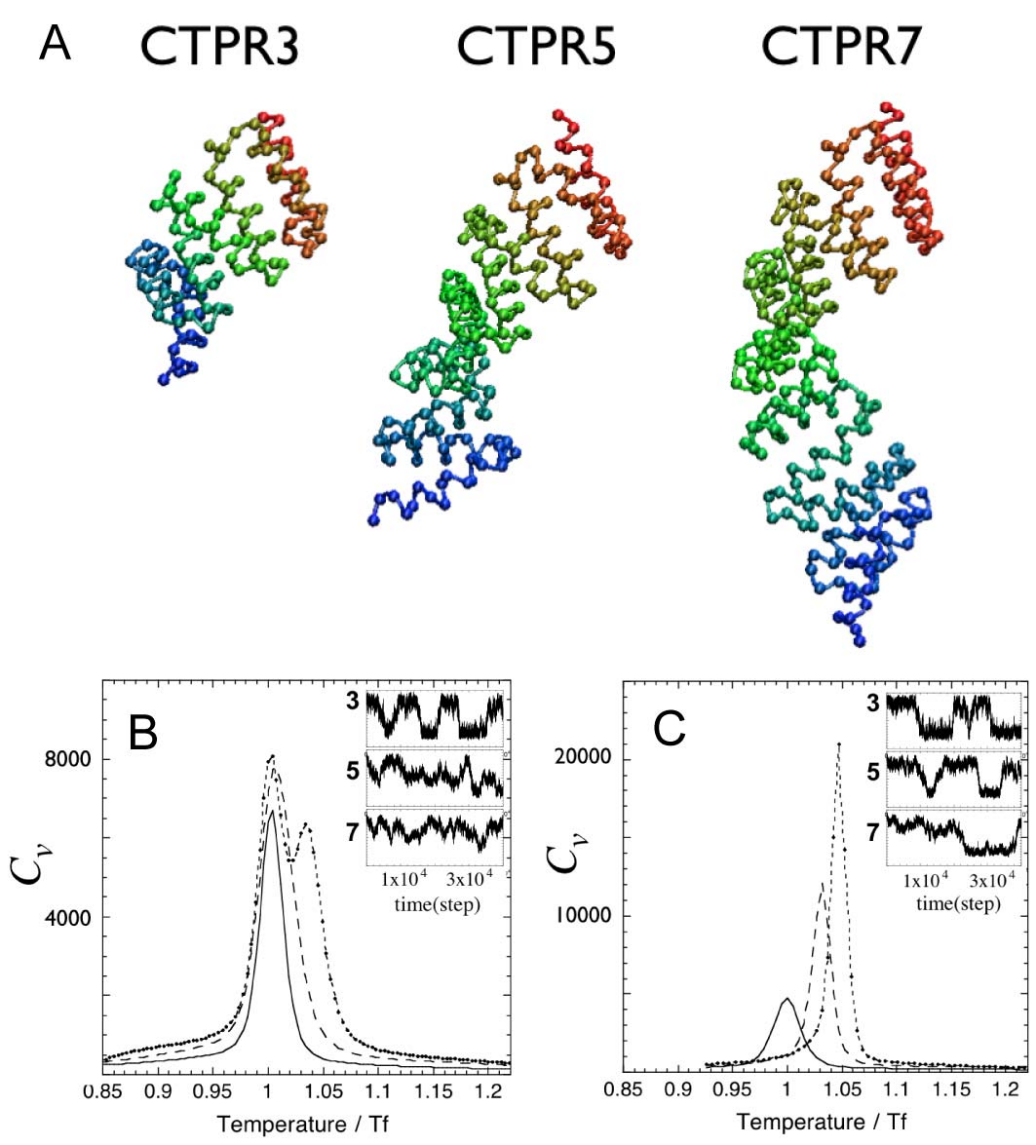
Molecular dynamic simulations on the folding of TPR proteins using a perfectly funneled topological potential. Representative structures of the native state of TPR proteins with 3, 5, or 7 repeats obtained after simulated annealing. (A) Heat capacity as a function of temperature for CTPR proteins of different number of repeats. The lines correspond to the data for proteins with 3 (solid), 5 (dashed), and 7 (dot-dashed) repeats. Inserts: raw data of the order parameter (Q) as a function of time from representative trajectories close to their respective *T_f_*. (B) Heat capacity as a function of temperature as in b) but for strictly homogeneous set of TPR proteins.

To illustrate the ideas, we simulate the folding of consensus TPR proteins (CTPRs) of different lengths. This is a good test case since high resolution structures are available, making it possible to evaluate the local energetic frustration of the protein [28]. These quantitative measures of frustration show that CTPR is unusually unfrustrated, justifying the use of perfectly funneled models to capture folding. Also, experiments have been already successfully interpreted with a simplified Ising-like description [7], which is also unfrustrated. It is worth noting that even though the sequence of each of the repeats in the consensus protein is the same, there are subtle structural differences that influence the number of contacts made by each repeat, particularly on the end units [33]. The major structural difference is present at the C-terminal repeat which contains an additional ‘capping’ helix. Based on the crystal structure of the 3 repeat protein CTPR3 [33] we constructed a series of proteins for which the terminal repeats are kept constant and the middle one is repeated from 1 to 6 times, thus constructing for computational study a family of proteins that have between 3 and 8 repeats. This family has been realized in the laboratory by Regan et al. [7]. Simulated annealing runs show that the constructed proteins converge to the TPR fold, as expected for this topology-based potential (Figure 4A).

Constant temperature runs were carried out at various different temperatures for each protein and the thermodynamic folding parameters were extracted by weighted histogram analysis, using the number of native contacts (Q) as an order parameter [34]. We noted that as the number of repeats increases, higher temperatures are needed to reach the unfolded state, that is, the proteins are more stable (Figure 4B). The shorter proteins display a single peak in the heat capacity as a function of temperature. This peak becomes broader and moves to higher temperature as the protein grows larger (Figure 4B). For proteins longer than six repeats a second peak can be distinguished. The shorter proteins conform more closely to a phenomenological two-state like behavior in which at any time mainly the fully folded or fully unfolded configurations are present. The decrease in cooperativity with increasing length is expected from the analytical model presented above only if the energetic parameters for the repeats are not homogeneous. To test this, we deleted the terminal repeats and strictly conserved the central ones, thus having a set of fully homogeneous proteins of increasing length. Following the same simulation and analysis protocol as before, we note that as the number of repeats increase, higher temperatures are needed to reach the unfolded state, but in this case the peak of heat capacity as a function of temperature becomes sharper (Figure 4C). Thus, as predicted for a strictly homogeneous protein, both stability and cooperativity increase with length.

**Figure 5:**
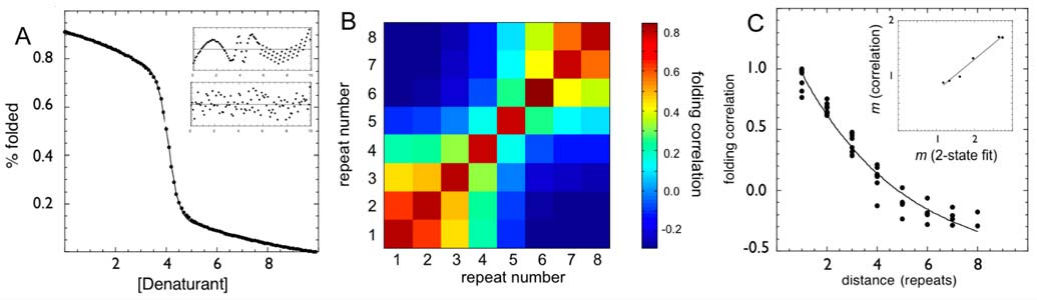
Folding simulations using a perfectly funneled topology-based model. (A) Fraction folded as a function of denaturant calculated from the simulation of a three repeat protein CTPR3. The line corresponds to the best fit to a two-state folding approximation. Inserts: top: residuals of the fit in the main figure, and residuals if 5% noise is added to the data before fitting. (B) Folding cross correlation of the repeats of CTPR8 calculated from the simulations near the folding temperature. (C) Dependence of the folding cross-correlation with distance separation as calculated from the MD simulations. The line is the best fit to an exponential decay. Insert: *m*-values calculated from the two-state approximation and calculated from the folding cross-correlation function.

To qualitatively compare the simulation results to the usual experimental denaturations, we linearly scaled the simulations to a percentage folded vs. denaturant and fitted the results with the linear free energy model of a two-state transition that is typically employed for experimental data analysis [32] (Figure 5A) (see Text S1). Even for the shorter proteins the residuals of the fits show deviations, but these deviations would usually go unnoticed experimentally since a 5% error in the signal easily overcomes the effects of the low populated, high free energy, intermediates (Figure 5A, inset). Taking into account the two-state fit as a reasonable approximation, the extracted stability and cooperativity parameters shows a linear relationship expected from the Ising description presented above (not shown).

In the previous section we showed that the folding cooperativity may be interpreted as a function of the folding correlation of the individual repeating units. In order to quantify these results and measure the correlation length of the simulated CTPR proteins, we computed the cross correlation of the folding of each repeat with every other, for each of the proteins (see Text S1). Figure 5B shows the cross correlation matrix for the longer 8-repeat protein, where two main correlation ‘domains’ are distinguished, centered towards the topological ends. The correlation length was extracted by fitting the cross correlations evaluated for all the proteins with the expected exponential decay in distance separation (Figure 5C). The absolute value of the correlation length is 2.9±0.3, roughly 3 repeats. Thus, it seems likely that as the number of repeats increases above the correlation length, the protein can ‘split’ into separate folding domains with a relatively low energy cost. The simulations show that the new peak in the heat capacity plot indeed does appear when the protein sequence is more than two times the correlation length, and that it corresponds to the folding of the new ‘domain’ formed by the additional repeats. Moreover, the overall cooperativity *m*-value calculated from the cross-correlations corresponds to the one fitted from the two-state approximation described above (Figure 5C insert).

### Extracting the Folding Parameters from the Molecular Dynamics Simulations

**Figure 6:**
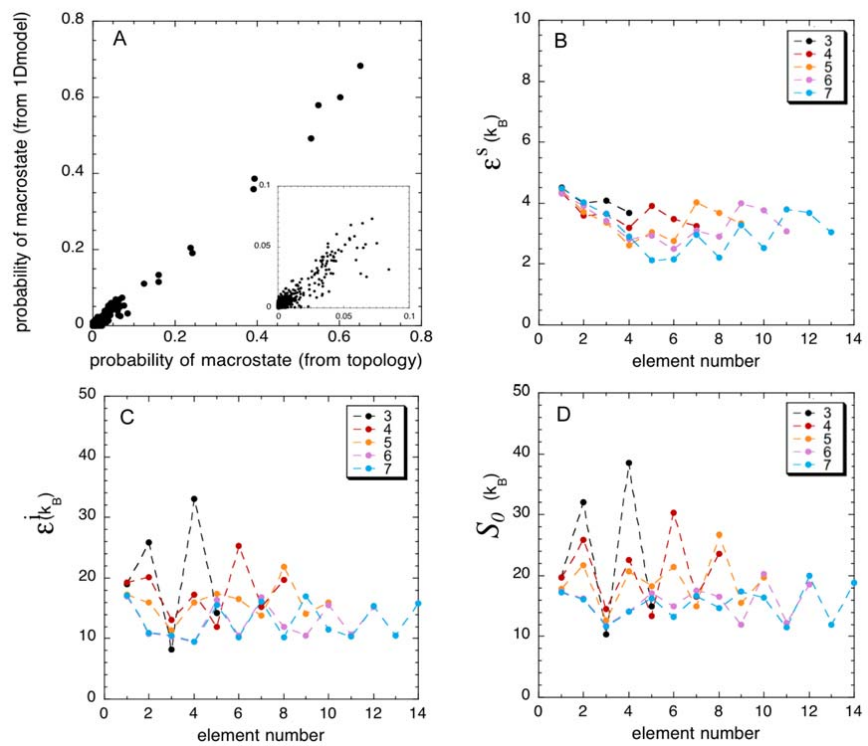
Application of the coarse-grained model to extract local information from the topology-based folding simulations. (A) The probability of occurrence of every possible folding macro-state at 11 different tempratures near *T_f_* extracted from the simulations (x-axis) or computed with the coarse-grain model (y-axis) after fitting the elementary parameters. (B–D) Values for the elementary parameters extracted from fitting the conformational distribution in every macro-state of every protein from the topological folding simulations to the coarse-grained model.

The residue level folding simulations qualitatively agree with the results from the analytical treatment of the one-dimensional reduced model that represents repeating elements as two-state objects. For quantitative analysis we developed a method for extracting the internal energetic parameters of each folding unit and its interactions. To do this we compute from the molecular dynamics trajectories the probability of occurrence of each possible folding macro-state as a function of temperature. The parameters of the reduced model are then fitted to reproduce these distributions for the residue-level simulations, by minimizing the Kullback-Leibler divergence between the model probabilities (*p_m_*) and those extracted from the simulation data (*p_s_*), 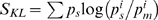. To define the totality of possible folding states and count them, one must first define boundaries between the folding elements. It is important to bear in mind that given the repeating patterns of repeat-proteins, this choice of division into subunits is not *a priori* unique. One natural choice is to define the folding element as the smallest strictly repeating unit in sequence, which corresponds to a whole TPR repeat. We analyzed the ensemble of trajectories using this definition, assigning a folding macro-state to each snapshot (from 000 to 111 in case of CTPR3), but found that the non-linear fitting was inconsistent and highly sensitive to the choice of initial parameters. This suggests that there are multiple minima to the error function. Similar inconsistencies were also observed for the longer proteins, suggesting that this initial choice of a quasi-independent ‘folding element’ was incorrect in the sense that the analytical model cannot describe the statistics of the low free energy ensembles of the topology-based model. Looking more carefully at the sampled structures it became apparent that each repeat can better be further decomposed into smaller folding units, namely two (or three in the case of C-terminal repeats) α-helices along with the interfaces between them. Since the present pure topology based model is biased to form α-helices because of the dihedral constraints and the low contact-order, we chose to assign the foldedness of each element based on the non-helical contacts formed. We use a cutoff value close to the separatrix of this projection to assign the microscopic state of each element (Figure S1). Kajander et. al. also employed a similar decomposition of the TPR-protein individual α-helices in a homogeneous Ising-like description of these proteins [7]. Using this choice of fundamental element, a CTPR3 protein can occupy any of 32 states (00000 to 11111), and the populations of these can be well described with the one-dimensional model (Figure 6A). We note that while the fitting method emphasizes the low free energy states, even the macro-states with very high free energy are well described, indicating that the model accounts well even for low populated folding intermediates.

The presented method pins down values for the elementary folding parameters of the coarse grained Ising description, shown in Figure 6. The individual intra-element entropy is about 20 *k_B_* with a compensating internal energy in the same range. Thus, at the folding temperature (*T* = *T_f_* = *1*), the free energy of each unit is near zero or slightly positive, that is to say, a single repeating unit is unstable by itself. In contrast, the interaction energy is highly favorable, about 3 *k_B_T*. The recurring zigzag patterns of the plots of Figure 6 indicates that the model indeed captures differences between the interactions of elements internal to one TPR-repeat and those of inter-TPR repeats, as expected from the difficulty to assign the folding unit to the whole TPR repeat. We note that for longer proteins the interaction energy decays for the units located towards the center. Apparently this is the location where ‘cracks’ are more likely to occur, as observed directly from examining configurations sampled in the molecular dynamics (Figure 5B).

**Figure 7:**
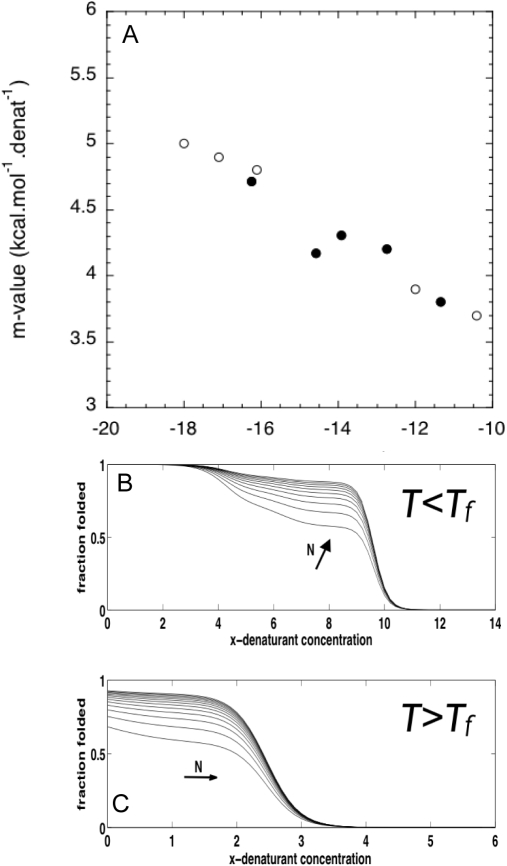
Application of the coarse-grained model to predict the chemical denaturant induced unfolding. (A) The computed free energy in water and *m*-value for the coarse grain model with the elementary parameters from Figure 6, and *T* = 0.9147 *T_f_*, α = 1.7 (closed circles), and the experimentally determined values (open circles). (B) Folding curves predicted for TPR proteins of different lengths (from 5 to 30), at *T* = *0.9 T_f_*. (C) Folding curves predicted for TPR proteins of different lengths (from 5 to 30), at *T* = 1.1 *T_f_*.

The value of the parameters for elementary interactions in the analytical model were determined from temperature denaturations of the topologically based model, but the values should be applicable to analyze any denaturation method. We further applied the analytical model to analyze the chemical denaturation behavior of the TPR-repeat protein family, and quantitatively compare the predicted parameters to the experimentally observed values. In making this mapping, there are two input parameters in the analytical treatment of chemical denaturations that need to be determined, the experimental folding temperature *T_f_*, and the susceptibility to denaturant parameter α*_j_*. We first treat these as free parameters and fit their values to minimize the differences in both the *m*-value and the ΔG_water_ to the experimentally measured ones. Figure 7A shows the close quantitative agreement between the experiments and values obtained from the analytical model. For the free parameters, the values we recover are *T = 0.91 T_f_*, and α*_j_* 1.7 *k_B_ [D]^−1^*. We can crudely compare this with the apparent folding temperature of CTPR3 (*∼355 K*) (http://www.yale.edu/reganlab) and the temperature at which the denaturations were actually performed (*∼298 K*), then *T = 0.84 T_f_*. In principle, the susceptibility can be estimated from the difference in solvent accessible surface area (SASA) between the fully folded and the fully unfolded states [35]. The *m*-value estimated from the crystallographic structure of CTPR3 is *1.5 kcal mol^−1^ [GuHCl]^−1^*. With this value, α*_j_* is estimated to be 0.7 (eq. 6), for an homogeneous repeat-protein, a 2.5 fold difference from the value recovered from the analytical model. This difference is likely to arise from the non-additivity of the natural interaction energetics, that are not explicitly treated in the topological model from which the parameters for the analytical model were derived (Figure 6). This parallels the behavior of activation free energies which are underestimated in purely additive models [36].

We can further apply the coarse-grained model to predict the experimental behavior of longer TPR proteins, at any experimental conditions. As examples we show the predicted folding curves for the chemical denaturation of proteins between 5 and 30 identical TPR repeats (Figure 7). If the temperature is lower than *T_f_*, it is directly observed that the proteins spontaneously populate partially folded species over a broad range of denaturant concentrations (Figure 7B). On the other hand, if the temperature is slightly higher than *T_f_*, the proteins are readily susceptible to denaturant, and even partially folded species of the shorter proteins are populated at zero denaturant concentration (Figure 7C). We note that this thermodynamic behavior has been experimentally observed in natural repeat-proteins from both the ANK and TPR families [18],[37].

**Figure 8:**
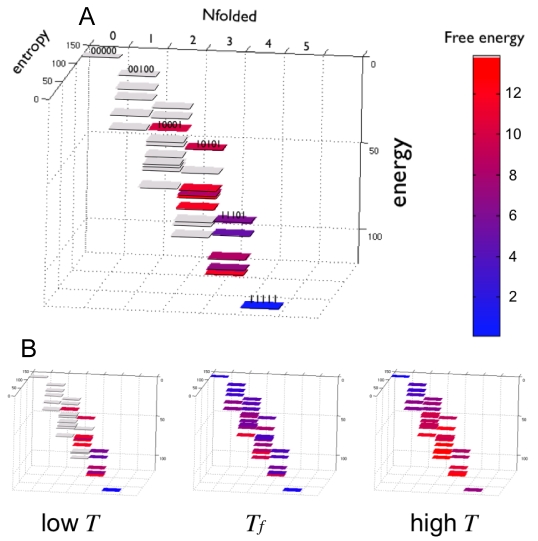
Folding energy landscape of repeat-proteins. (A) A 5-element repeat-protein folding landscape is shown. Each macrostate is depicted as function of the number of folded elements, their internal energy, and added entropy. The population of each possible macrostate is shown colored according to its free energy. Some of the macrostates are labeled according to the location of the folded elements from N to C termini (00000 fully unfolded, 11111 fully folded). (B) Change in the relative population of each configuration as the temperature changes.

Since the presented method recovers the values for all the parameters of the analytical model, we can compute the overall energy and entropy of every possible macro-state. The resulting repeat-protein folding free energy landscape is presented in Figure 8. We use for illustration the example of the shortest three TPR-repeat protein. The fully unfolded state is at the top of the funnel, a state that has the highest entropy and the lowest energy (Figure 8A). At the bottom, the fully folded state has the opposite contributions. In between there exist several discrete possibilities corresponding to the folding of different numbers of elements. Each of these ‘steps’ has a characteristic energy/added entropy balance, reflecting the fact that the protein is finite and inhomogeneous. The added entropy is a result of the underlying degrees of freedom, which we describe within the coarse grained model. The population of each of the states depends on this precise balance at any given temperature. At low temperatures, the fully folded states and states with 4 elements folded (or 1 ‘crack’ introduced) are mostly populated, while at high temperatures the fully unfolded and the states with one-element folded are populated (Figure 8B). Right at the folding temperature several intermediate species become populated, and this distribution need not be homogeneous (Figure 8B). Each of these intermediate states will contribute to the overall cooperativity and stability of the repeat-protein domain, and the spontaneous population of them might be functionally relevant, as discussed below.

### Experimental Observations

**Figure 9:**
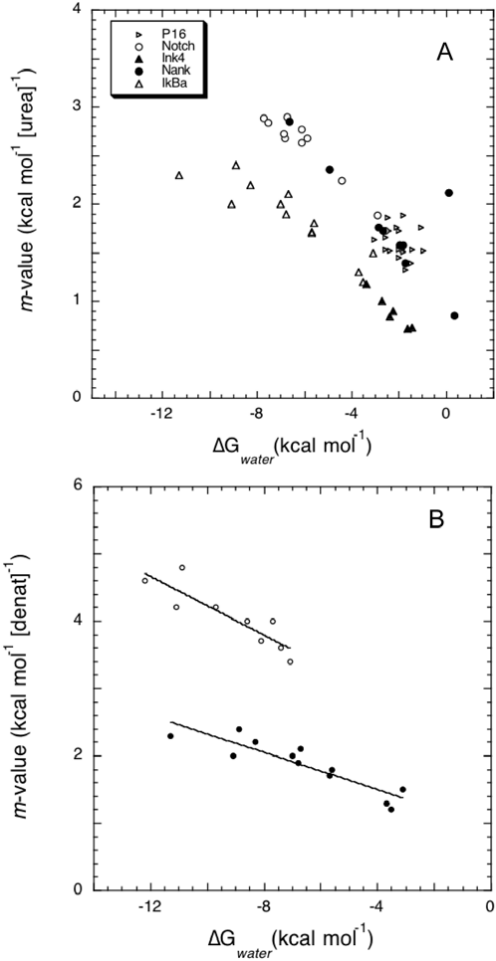
Experimental observations of the effect of single point mutants on the overall folding behavior. (A) Folding parameters determined by urea denaturation of various mutants of ankyrin repeat proteins, upon fitting to two-state models [8], [18], [38]–[40]. (B) Folding parameters determined by urea (closed circles) or guanidinum hydrochloride (open circles) denaturation of various mutants of IκB-α, upon fitting to two-state model [18].

Both the one-dimensional model and the native topology-based perfect funnel simulations presented above show a fundamental relationship between the stability and the cooperativity of the folding of repeat-containing proteins. The model predicts that single site mutations will affect both of these global folding descriptors simultaneously. In order to test this prediction, we collected data from the literature for several independent folding experiments performed on distinct repeating proteins [8], [18], [38]–[40]. As mentioned earlier, the folding profiles for these relatively short proteins (between 4 and 7 repeats) can usually be approximated by a two-state folding model, so a single *m*-value and the free energy in water is usually reported. Figure 9A shows the experimental folding parameters for single amino acid mutations performed on several ankyrin-repeat proteins. Indeed, a steep relation is found between the two global folding descriptors, even when the proteins analyzed have different numbers of repeats, the mutations are not necessarily analogous, and are distributed along different units. The slope of the *m vs.* Δ*G* plot is constant for each set of mutants, and we attribute the offset between them to the fact that the proteins themselves and the experimental conditions under which they were measured are not identical, and are expected to change the definition of ‘native’ stability. This indicates that the (de)stabilizing effect of a single mutation can be explained only in terms of the alteration it causes to the interactions of the repeat with its immediate neighbors, modifying the cooperative behavior of the whole system, as predicted from the analytical model (Figure 3C).

When the same mutations done on a single protein are analyzed by denaturation with urea or guanidine hydrochloride, the extracted folding parameters may differ, an effect often attributed to differences in residual structure of the unfolded state or changes in the solution conditions (*i.e.* ionic strength) during denaturation. Figure 9B shows that the linear relationship between ΔG_water_ and *m*-value still holds when different denaturants are used to probe the folding transitions [18]. The change in slope of the plot is directly related to the change in the denaturation midpoint (*x_c_* in the model), that is expected to change with denaturant. We further note that the width in the distribution of the data points is different for each denaturant. This observation is well modeled by a change in the repeat interaction parameter (ε*^S^*), suggesting that the solution conditions modify the interfaces between the repeating elements as well as the folding of the elements themselves. In principle, the detailed thermodynamic contribution of each ‘unit’ can be extracted from experiments by analyzing the effect that analogous mutations along the repeating domain cause on the global folding parameters. In the large N limit, the slope of the *m_x_* vs ΔG*^x^*_water_ plot is 
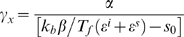
. In order to decompose the contributions from these energy scales, the effects on the cross-correlation of the folding elements of several independent mutations should be evaluated. Unfortunately, in the laboratory, temperature unfolding of these proteins is often irreversible owing to aggregation, limiting the strict quantification of the thermodynamic profiles at this stage.

### Concluding Remarks

Repeat proteins constitute excellent biological systems for which simple physical folding models can be directly evaluated. In contrast to globular domains they lack interactions distant in sequence and have fairly regular architectures. This features greatly simplifies the coarse-graining of the interactions internal to and between the folding elements. The Ising model was originally conceived as a description of magnetism in crystalline materials, and has been applied to model phenomena as diverse as the freezing and evaporation of liquids, the behavior of glassy substances, flocking birds, neural networks, and the folding of protein secondary structures. Recently this type of model has been also applied to describe the experimental folding of repeat proteins (for a recent review see [2]). Here, we have shown how some puzzling features of the folding of repeat proteins when analyzed viewing them as single cooperative units like globular proteins in fact follow from their one dimensionality. The finite correlations intrinsic to one dimensionality yield a direct relationship between stability and cooperativity of the folding transitions. We have shown how this relationship arises, and how this relation changes in different parameter regimes (Figures 1–3). Depending on the balance between the folding energy of each unit and the interactions with neighbors, very different behavior can be obtained when the whole repeating array is evaluated. In which parameter space do natural repeat-containing proteins exist? It is experimentally observed that single-site mutations affect both stability and cooperativity simultaneously in the same direction, and we show this is most likely a consequence of changing the interaction energy between repeats. Moreover, from the large absolute values that the single-site mutations exert on both stability and cooperativity, we speculate that the natural wild-type proteins may well be ‘poised’ at particular ratios of inter/intra element energetics that allows small local perturbations to yield large effects. The linear relationship between these global parameters is expected to break down at extreme ratios (Figures 1–3), either when the correlation length spans the whole repeating array or the interactions between some elements is low enough to ‘decouple’ them. In line with this prediction, a detailed series of experiments was recently presented by Street et al showing that the *m*-value change upon destabilization saturates when sufficiently large destabilizations are probed [17]. A local effect is felt globally because the near interactions play extraordinarily large roles in stabilizing the repeats, and weak biases can tip the balance to complete folding [16],[41]. It has been observed that single substitutions that affect local biases (such as helix propensity), exert profound effects on the overall folding of these domains [18],[40],[42].

Modeling folding cooperativity is functionally relevant and was previously addressed in lattice and off-lattice models of globular proteins [43],[44]. In the model presented here, the folding cooperativity can be directly related to the correlation length of the repeating array, and this in turn is a function of the local interaction parameters. Taking the native TPR topology as an example, we have shown that the *m*-value derived from the 2-state approximation corresponds to the cooperative parameter derived from the one-dimensional description (Figure 5). Within the 2-state approximation it is the relative population of spectroscopically indistinguishable folding intermediates that affects the *m*-value determination. We found a correlation length of 3 repeats. It is probably not a coincidence that this seems to be also the smallest functional unit for TPRs, found via bioinformatic analysis [45]. This correlation length has also been directly measured by NMR [46]. As the protein grows larger, the repeating array is more likely to tolerate ‘cracks’ and the folding at either end may become anticorrelated. If the protein is sufficiently large the energy of the fully folded state needs to be so favorable (or the temperature so low), that partially cracked species will become dominant under physiologically relevant conditions. There are at least three examples for which partially folded species have been experimentally characterized in repeat-proteins [18],[19],[42]. The largest repeat protein characterized to date is D34 a 12-ankyrin repeat fragment of the AnkyrinR protein. This fragment populates partially folded species and subtle perturbation of the repeat energetics has been shown to ‘mold’ the ensembles [19].

Repeat-proteins, as any other natural protein, are unlikely to function alone, without interacting with other macromolecules. We envision that many more natural repeat-proteins will show this relative ‘instability’ of certain parts of the array, since it may be functionally indispensable to undergo folding transitions upon binding, as in the case for IκBα and Notch [47],[48]. Our observations describe how changing the stability of a single repeating element (by posttranslational modifications or binding of other macromolecules) would affect the behavior at a distant site, providing a coupling mechanism that can transmit allosteric signals to long distances within a single repeating array.

## Methods

### Numerical Methods

The numerical evaluations of the coarse-grained model as well as the fitting procedures were performed using in-house Matlab scripts, available upon request.

### Perfectly Funneled Folding Simulations

The simulations were performed with a topological C〈 - based Gõ model, that takes into account only interactions present in the native structure and therefore does not include energetic frustration. Details of the model have been previously described when simulating the folding of other repeat-containing proteins [15]. Here, the high resolution structures of CTPR3 protein was used as a starting point (PDB id code: 1NA0). The parameters (contacts and dihedrals) of the central TPR repeat of this protein were repeated from 1 to 6 times, and the end-repeats were conserved, generating a family of 3 to 8 CTPR-like proteins. For generating the potential for fully homogeneous TPR-proteins, only the central TPR repeat of CTPR3 was repeated 3, 5, or 7 times. Further details can be found in the supplementary material (Text S1 and Figure S1).

### Graphical Representations

Proteins were visualized using VMD [49]. All other graphical representations were done using Matlab and Kaleidagraph.

## Supporting Information

Figure S1 Folding free energy surfaces of individual elements from a repeat-protein. The free energy surfaces of the elements of the simulated CTPR3 protein are plotted as a function of the intra-element native contacts (*Q_i_*) versus the total number of native contacts (*Q_t_*). Each element is defined as the set of all non intra-helical native contacts each TPR helix makes. The color scale represents the free energy calculated at the folding temperature and is in units of *ε*. Mainly two low free energy states are distinguished in every case. The line makes the cut-off value used to assign the folding status of each element (see main text).
(2.57 MB TIF)

Text S1 Supplementary text. Text contains details of the methods used.
(0.09 MB DOC)
