# Supplementary figures and images for: The Energy Landscapes of Repeat-Containing Proteins: Topology, Cooperativity, and the Folding Funnels of One-Dimensional Architectures
Source: PLoS Comput Biol. 2008 May 16;4(5):e1000070. doi: 10.1371/journal.pcbi.1000070 (PMC2366061; doi:10.1371/journal.pcbi.1000070)

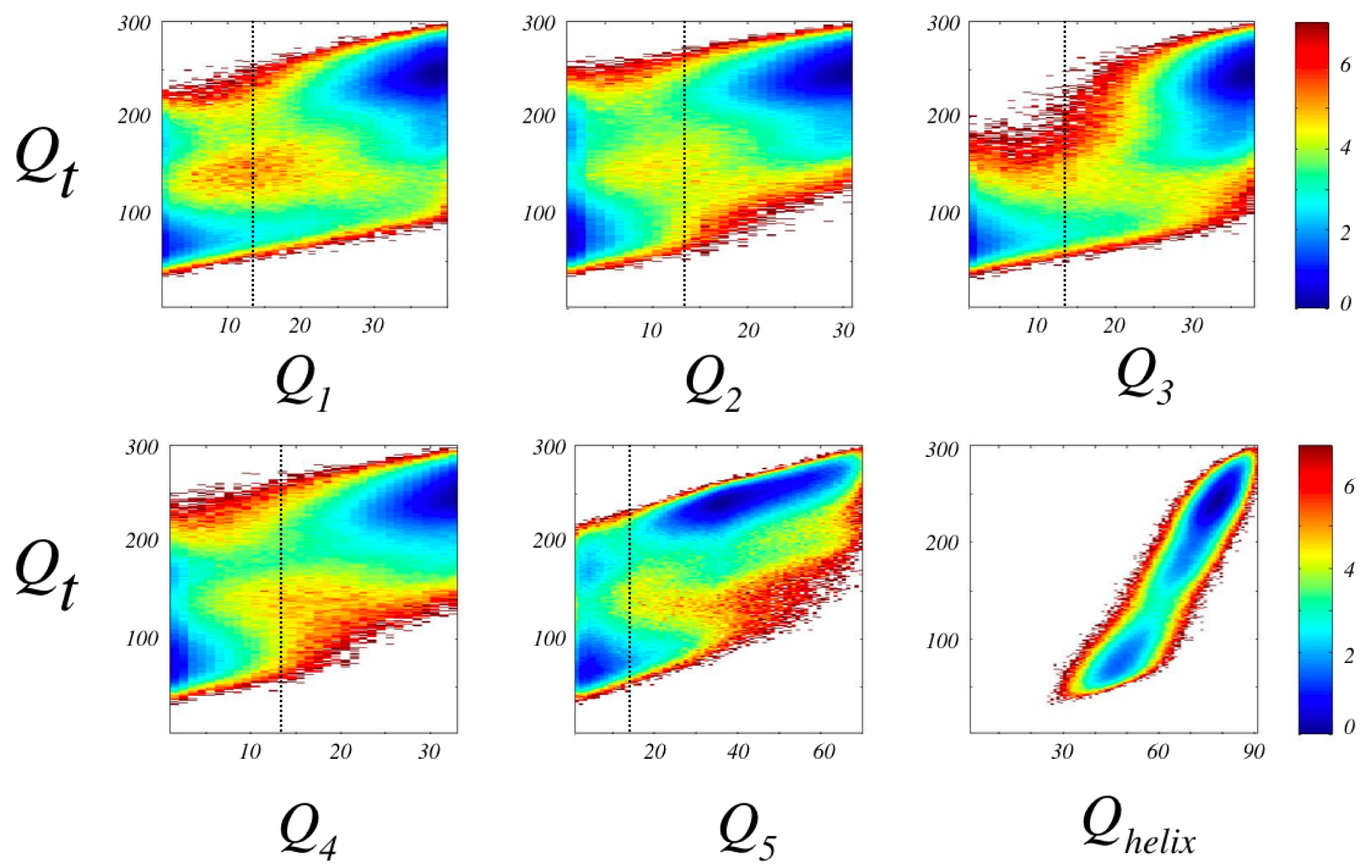

Supplement: Figure S1 — Folding free energy surfaces of individual elements from a repeat-protein. The free energy surfaces of the elements of the simulated CTPR3 protein are plotted as a function of the intra-element native contacts (Qi) versus the total number of native contacts (Qt). Each element is defined as the set of all non intra-helical native contacts each TPR helix makes. The color scale represents the free energy calculated at the folding temperature and is in units of ε. Mainly two low free energy states are distinguished in every case. The line makes the cut-off value used to assign the folding status of each element (see main text). (2.57 MB TIF) [file pcbi.1000070.s001.tif]
