# Supplementary material for: The Energy Landscapes of Repeat-Containing Proteins: Topology, Cooperativity, and the Folding Funnels of One-Dimensional Architectures
Source: PLoS Comput Biol. 2008 May 16;4(5):e1000070. doi: 10.1371/journal.pcbi.1000070 (PMC2366061; doi:10.1371/journal.pcbi.1000070)
Supplement: Text S1 — Supplementary text. Text contains details of the methods used. (0.09 MB DOC) [file pcbi.1000070.s002.doc]

Supplementary information

The Energy Landscapes of Repeat-Containing Proteins:

Topology, Cooperativity and the Folding Funnels of One-Dimensional Architectures

D.U. Ferreiro, A.M. Walczak, E.A. Komives and P.G. Wolynes

**Folding Simulations on Perfectly Funneled Landscapes**

The folding simulations on perfectly funneled landscapes were performed with an off-lattice Cα - Gõ model [1]. The energy function only takes into account interactions present in the native state and therefore does not include any energetic frustration from non-native interactions. Based on a crystallographic structure, an interaction between a pair of residues (*i, j*) is defined if the distance between the Cα atoms is smaller than 8 Å or the distance between any side chain heavy atoms of the two residues is smaller than 4 Å. Native contacts between pairs of residues (*i, j*) close in sequence |*i - j*| < 4 were discarded from the native contact list because contiguous residues already interact through the bond, angle and dihedral terms also included in the energy function (see below). Each residue is represented by a single bead centered in its Cα position, and adjacent beads are strung together into a polymer chain by means of a potential encoding bond length and angle constraints. The secondary structure is encoded in the dihedral angle potential and the non-bonded (native contact) potential. The interaction energy *U* for a given protein conformation Γ is given by:


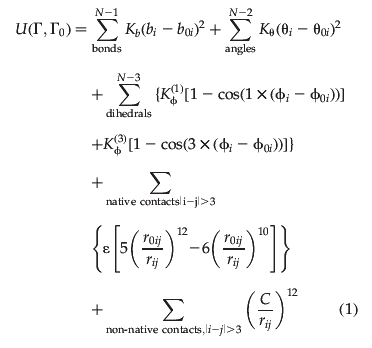


In this equation, *b_i_*, θ*_i_*, and φ*_i_* are the *i*th virtual bond length between *i*th and (*i* + 1)th residue, the virtual bond angle between (*i* -1)th and *i*th bonds, and the virtual dihedral angle around the *i*th bond, respectively. The parameters *b_oi_*, θ*_oi_*, and φ*_oi_* stand for the corresponding variables in the native structure. All native contacts are represented by a 10-12 Lennard Jones potential without any discrimination between the various types of interactions. The *r_ij_* and *r_0ij_* are the Cα-Cα distance between the contacting residues *i* and *j* in conformation Γ and Γ_0_ (the PDB structure), respectively. In the summation over non-native contacts, *C* (= 4.0 Å ) parameterizes the excluded volume repulsion between residue pairs that do not belong to the given native contact set. Here, all the temperatures and energies are reported in units of ε. For he other parameters, we use similar values to those used in several other folding studies [2-4] *K_b_* = 100.0, *K*_θ_ = 20.0, *K^(1)^*_φ_ = 1.0, *K^(3)^*_φ_ = 0.5, ε= 1.0.

We used molecular dynamics to explore the phase space. We employed the simulation package AMBER at constant temperature, except for the initial simulated annealing runs. For each model, between 100 and 200 constant temperature simulations were made and combined using the WHAM algorithm [5] to generate a heat capacity profile as a function of temperature and a free energy F (Q) as a function of the reaction coordinate Q [2]. For the calculations of the probability of contact formation, a contact was considered to be formed if the distance between the Cα atoms is shorter than γ times their native distance *r_0ij_*. It has been shown that the results do not strongly depend on the choice made for the cut-off distance γ [3]. Here we used γ = 1.25.

**Inter-repeat folding correlation**

The folding cross correlations were calculated from the molecular dynamics trajectories by analysing the occurence of every possible folding macro-state of a repeat protein. For each snapshot of the folding trajectories performed at constant temperature close to T_f_, the number of native contacts formed by each repeat (*Q_i_*) was counted. The repeat is assign as folded, $s_{i}$=1, if the number of native contacts is bigger than a cuttoff value which was set close to the separatrix of the *Q* coordinate that distinguishes between the folded and unfolded basins (see Figure S1). Otherwise, the repeat is assign to be unfolded, $s_{i}$= -1 .

The folding cross correlation between the elements *i,j* is defined by:

As an example, the values obtained for each *C_ij_* of the 8-repeat protein are displayed in Figure 5B of the manuscript.

**Two-state analysis of the folding transitions**

In order to qualitativelly compare the folding trajectories obtained from the simulations on perfectly funneld landscapes with the usual experimental determinations of protein stability, we linearly scaled the change in energy of the protein (*E*) with temperature (*T*) obtained from the WHAM analysis, to a ‘fraction folded’ (*f*) vs denaturant (*D*) scale:

were *T_1_* and *T_2_* are reference temperatures were the folded ensemble has an average energy *E_N_,* and the unfolded ensemble has and average energy *E_U_* , respectivelly. *λ *was set to 10 M.

The folding curves were fitted to a two-state transition [6]:

*f* = (a_1_ + p_1_[D]) + (a_2_ + p_2_[D]) exp(-(*∆G* - *m* [D]/RT) / 1 + exp(-(*∆G* - *m* [D]/RT)

where p_1_ and p_2_ are the pre and post transition baselines, a_1_ and a_2_ their corresponding y-intercepts, *∆G* is the folding free energy in water and *m* is the cooperativity parameter.

References

1. Clementi C, Nymeyer H, Onuchic JN (2000) Topological and energetic factors: what determines the structural details of the transition state ensemble and "en-route" intermediates for protein folding? An investigation for small globular proteins. J Mol Biol 298: 937-953.

2. Cho SS, Levy Y, Wolynes PG (2006) P versus Q: structural reaction coordinates capture protein folding on smooth landscapes. Proc Natl Acad Sci U S A 103: 586-591.

3. Clementi C, Jennings PA, Onuchic JN (2000) How native-state topology affects the folding of dihydrofolate reductase and interleukin-1beta. Proc Natl Acad Sci U S A 97: 5871-5876.

4. Ferreiro DU, Cho SS, Komives EA, Wolynes PG (2005) The energy landscape of modular repeat proteins: topology determines folding mechanism in the ankyrin family. J Mol Biol 354: 679-692.

5. Swendsen RH (1993) Modern Methods of Analyzing Monte-Carlo Computer-Simulations. Physica A 194: 53-62.

6. Pace CN (1986) Determination and analysis of urea and guanidine hydrochloride denaturation curves. Methods Enzymol 131: 266-280.
